# Endoscopic submucosal dissection of a huge lipoma of ileum assisted with in-vivo traction

**DOI:** 10.1055/a-2604-8011

**Published:** 2025-06-26

**Authors:** Xin Sun, Ziwen Zhao, Ruyuan Li

**Affiliations:** 1 91623 Department of Gastroenterology, Qilu Hospital (Qingdao) of Shandong University, Qingdao, Shandong, China; 2 Department of Gastrointestinal Surgery, The No. 4 Municipal Hospital Affiliated to Shandong First Medical University, Jinan, Shandong, China

A 46-year-old man with abdominal pain underwent colonoscopy, which revealed an ileal tumor. A pelvic-enhanced CT showed a lipoma of the ileum, measuring approximately 3.2 cm by 2.3 cm. Colonoscopy displayed a giant mass obstructing the small bowel lumen ( Fig. 1 **a** ). The lesion was located near the cecum, and the base of the mass could not be clearly exposed. To better visualize the base of the mass, we applied two elastic rings to draw the lipoma into the colon. A clip with one elastic ring (Patent number: ZL2020 2 0016729.9, China) was inserted into the lumen through the biopsy channel of the scope and fixed onto the lipoma ( Fig. 1 **b** ). The second and third clips grasped the ring and anchored it to the colon. A second traction ring was placed using the same method. The root of the lipoma was shaped into a triangular configuration ( Fig. 1 **c** ). The tissue clips occluded the base of the lesion, blocking blood flow. Epinephrine and methylene blue saline (1:10,000) were injected into the submucosa at the root of the lesion to completely elevate it. A C-shaped mucosal incision was made using a DualKnife (KD-650Q, Japan, Olympus Company) ( Fig. 1 **d** ). The entire giant lipoma was dissected en bloc ( Video 1 ). A hemostatic forceps (FD-410LR, Olympus Company) was used to manage any vascular stump bleeding, and the wound was sutured with tissue clips (Nanjing Minimally Invasive Company). There were no complications, such as perforation or delayed hemorrhage at the wound site. The size of the tumor was determined to be 5.0 cm by 6.0 cm ( Fig. 1 **e** ), and pathology confirmed it as a lipoma.

**Fig. 1:**
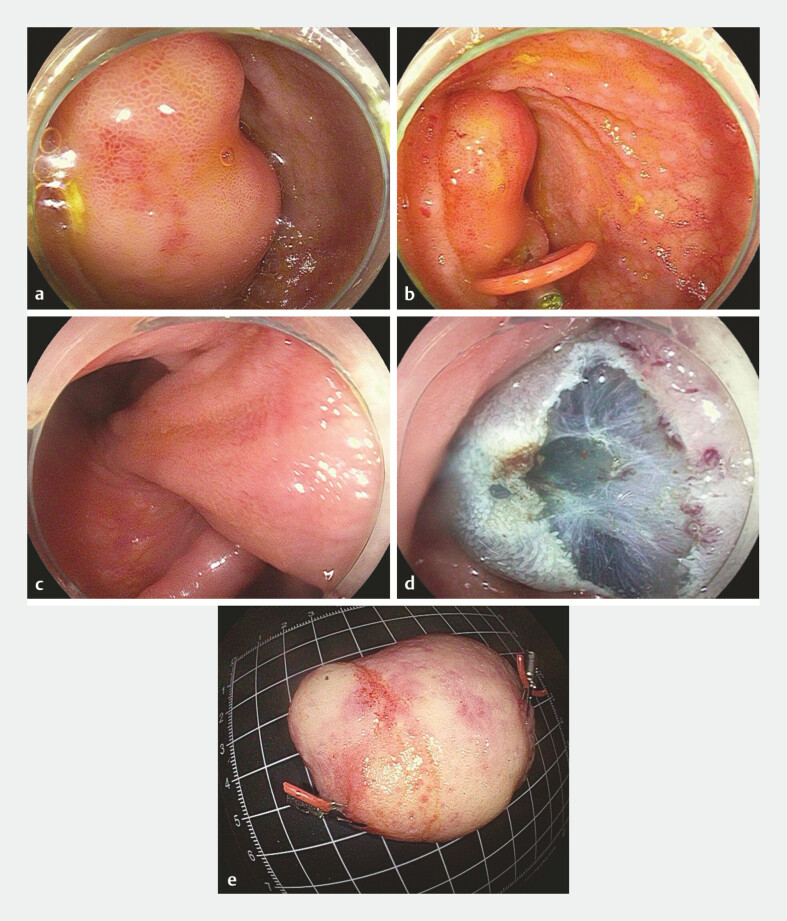
**a** Colonoscopy revealed a giant mass obstructing the small bowel lumen. **b** A clip with one elastic ring was inserted into the lumen through the biopsy channel of the scope and fixed onto the lipoma. **c** The root of the lipoma was shaped into a triangular configuration. **d** A C-shaped mucosal incision was made using a DualKnife. **e** The size of the tumor was determined to be 5.0 cm by 6.0 cm.

Endoscopic submucosal dissection of a huge ileal lipoma assisted with in-vivo traction.Video 1

The lipoma of the ileum can cause intestinal intussusception. Large-sized lipomas may have malignant potential or be difficult to distinguish from other malignant tumors 1 . Surgical resection, including laparoscopic surgery, is a common treatment for lipomas of the small bowel 2 . Small bowel tumors present a higher degree of complexity for endoscopic resection, and surgical treatment is sometimes required in cases of incomplete resection or major adverse events (mAEs). However, advances in small bowel evaluation and therapeutic endoscopy will increase detection and resection rates of small bowel tumors, reducing the need for surgical intervention in often complicated clinical scenarios 3 . We successfully performed ESD of the lipoma of the ileum with in-vivo traction assistance. Our study provides a novel technique for the treatment of small bowel lesions.

Endoscopy_UCTN_Code_TTT_1AQ_2AD_3AD

## Notes

Endoscopy E-Videos https://eref.thieme.de/e-videos
*E-Videos* is an open access online section of the journal *Endoscopy* , reporting on interesting cases and new techniques in gastroenterological endoscopy. All papers include a high-quality video and are published with a Creative Commons CC-BY license. Endoscopy E-Videos qualify for HINARI discounts and waivers and eligibility is automatically checked during the submission process. We grant 100% waivers to articles whose corresponding authors are based in Group A countries and 50% waivers to those who are based in Group B countries as classified by Research4Life (see: https://www.research4life.org/access/eligibility/ ). This section has its own submission website at https://mc.manuscriptcentral.com/e-videos .
